# Collaboration and knowledge integration for successful brain therapeutics – lessons learned from the pandemic

**DOI:** 10.1242/dmm.049755

**Published:** 2022-12-21

**Authors:** Maria Isabel Loza, Julija Hmeljak, Chas Bountra, James E. Audia, Sohini Chowdhury, Shannon Weiman, Kalpana Merchant, Maria-Jesus Blanco

**Affiliations:** ^1^ Center for Research in Molecular Medicine and Chronic Diseases (CIMUS), Pharmacology Department, School of Pharmacy, University of Santiago de Compostela, Health Research Institute (IDIS), Kærtor Foundation, 15706 Santiago de Compostela, Spain; ^2^ Disease Models & Mechanisms, The Company of Biologists, Bidder Building, Station Road, Histon, Cambridge CB24 9LF, UK; ^3^ Dorothy Crowfoot Hodgkin Building, Dorothy Hodgkin Road, University of Oxford, Oxford OX1 3QU, UK; ^4^ Flare Therapeutics, 215 1st Street, Cambridge, MA, 02142, USA; ^5^ The Michael J. Fox Foundation for Parkinson's Research, 111 West 33 Street, New York, NY 10120, USA; ^6^ Keystone Symposia, 160 U.S. Highway 6, Suite 201, PO Box 1630, Silverthorne, CO 80498, USA; ^7^ Northwestern University, 303 E Chicago Ave., Chicago, IL 60611, USA; ^8^ Atavistik Bio, 38 Sidney Street, Cambridge MA 02139, USA

**Keywords:** Brain therapeutics, Networking, Translation

## Abstract

Brain diseases are a major cause of death and disability worldwide and contribute significantly to years of potential life lost. Although there have been considerable advances in biological mechanisms associated with brain disorders as well as drug discovery paradigms in recent years, these have not been sufficiently translated into effective treatments. This Special Article expands on Keystone Symposia's pre- and post-pandemic panel discussions on translational neuroscience research. In the article, we discuss how lessons learned from the COVID-19 pandemic can catalyze critical progress in translational research, with efficient collaboration bridging the gap between basic discovery and clinical application. To achieve this, we must place patients at the center of the research paradigm. Furthermore, we need commitment from all collaborators to jointly mitigate the risk associated with the research process. This will require support from investors, the public sector and pharmaceutical companies to translate disease mechanisms into world-class drugs. We also discuss the role of scientific publishing in supporting these models of open innovation. Open science journals can now function as hubs to accelerate progress from discovery to treatments, in neuroscience in particular, making this process less tortuous by bringing scientists together and enabling them to exchange data, tools and knowledge effectively. As stakeholders from a broad range of scientific professions, we feel an urgency to advance brain disease therapies and encourage readers to work together in tackling this challenge.

## Teaser

**Summary:** This article summarizes the key points and action items that emerged from a virtual Keystone Symposium on advancing brain therapeutics.

## Introduction

Brain diseases are a significant cause of death worldwide and contribute significantly to years of potential life lost due to the increasing prevalence of mood, neurodevelopmental and behavior disorders in young people (Forlund et al., 2020; Olfson et al., 2015; Liu et al., 2020). Recent years have seen significant advances in drug discovery and development, and approaches and technologies in central nervous system (CNS) research (as discussed in detail in, for example, Forlund et al., 2020; Olfson et al., 2015; Bloem et al., 2021; Sinnige et al., 2020; Lüscher Dias et al., 2020; Dhanwani et al., 2022; Anderson et al., 2022; Baird et al., 2021; Clinical Outcomes Assessment Selection). Unfortunately, this progress has not been sufficiently translated into successful treatments. Obstacles such as limited understanding of heterogeneous CNS disease etiologies, lack of predictive preclinical models and inefficient drug delivery to the brain remain a challenge in the field (Morofuji and Nakagawa, 2020; Howes and Mehta, 2021). Taken together, these obstacles contribute to the high investment risk associated with brain drug discovery. In addition, the coronavirus disease 2019 (COVID-19) pandemic has exacerbated the impact of brain diseases on worldwide populations. For instance, there have been increased diagnoses of anxiety, depression and other mood disorders (Farooq et al., 2021; Guessoum et al., 2020), and disrupted access to routine care for chronic degenerative diseases of the CNS, such as Parkinson's disease (PD) and Alzheimer's disease (AD) (Helmich and Bloem, 2020; Mok et al., 2020).

Despite the disruption, the COVID-19 pandemic has provided us with a new outlook on how we can work together to accelerate drug discovery. This could help the community develop new drug discovery approaches in brain diseases and bring scientific and therapeutic advances to patients.

This Special Article expands upon a Keystone Symposia virtual panel discussion, organized by M-J.B., M.I.L. and K.M. (Box 1). We place the main highlights of the discussion in the wider context of translational neuroscience and discuss the critical progress needed to integrate diverse backgrounds and capabilities of patients, funders, preclinical researchers and clinicians to ultimately develop successful brain therapeutics. We propose a list of action items (Box 2) to facilitate this process.

**Box 1. Keystone Symposia Brain Therapeutics series**

The virtual panel ‘Brain Therapeutics: Lessons Learned from Pandemic Times’ revisited the topics and challenges discussed at the Keystone Symposium ‘Brain Therapeutics: Disruptive Technologies and Opportunities’ conference, held on 16-19 February 2020, in Santa Fe, NM, with the goal of bringing new insights to brain therapeutics from the COVID-19 pandemic.

During the 2020 in-person meeting, a roundtable discussion was held in which panelists and meeting participants explored various approaches and challenges to successful drug discovery in brain therapeutics, with deep, rigorous and passionate review of the current landscape. The workshop proved highly successful, producing innovative strategies to overcome current roadblocks and yielding a future vision to accelerate translational successes in the field of brain therapeutics.

Although the goal of this original workshop – to identify strategies and discuss key challenges in drug discovery for brain diseases – was achieved, the COVID-19 pandemic swept through the world soon after, significantly changing the landscape in which these solutions could be implemented and providing novel insights into drug discovery approaches.

This was the motivation for revisiting the panel discussion 18 months later in a virtual format, drawing in participants from around the globe to learn from past experiences and collaborate towards innovative approaches. The live event took place on 9 September 2021, and the recording is freely available on demand via a Virtual Keystone Symposia page. Highlights of the event and post-discussion Q&A session are published on the Keypoint blog.

We invite readers to consider participating in the upcoming Keystone Symposia meeting ‘Drug Delivery to the Brain: Challenges and Progress’, which will touch on many of the themes discussed during our virtual panel and in this article. The meeting will be held in person from 23 to 26 January 2023, and talks will be available for on-demand viewing.

**Panelists:**

**Maria Isabel (Mabel) Loza** is Professor of Pharmacology and Head of the BioFarma research group at the University of Santiago de Compostela, and is Trustee and Scientific Director of the Kærtor Foundation. Mabel has 25 years' experience in public–private partnerships in drug discovery, from which 16 new chemical entities have reached clinical trials, and specializes in accelerating the early drug discovery process to patient proof-of-concept studies.

**Maria-Jesus Blanco** is currently Vice President and Head of Chemical Sciences at Atavistik Bio, Cambridge, MA. Maria has more than 20 years of experience in the pharmaceutical industry, focusing on discovery and development of small-molecule and new chemical modalities in CNS disorders, oncology, chemical biology and translational research to bring meaningful therapies to patients.

**Kalpana Merchant** is Adjunct Professor of Neurology at Northwestern University Feinberg School of Medicine and is President and Chief Scientific Officer of TransThera Consulting Co. She has 25 years' experience in pharmaceutical drug discovery and development, and is Strategic and Scientific Advisor and Member of the Executive Scientific Advisory Board for The Michael J. Fox Foundation for Parkinson's Research. Kalpana provides consulting services to private investors, including Third Rock Ventures, Vomisa, start-up biopharma/pharma companies and life sciences technology companies.

**James E. Audia** represented the Chicago Biomedical Consortium during the panel discussion and is now a Founding Scientist and Executive Vice President of Drug Discovery and Early Development at Flare Therapeutics. He has more than 35 years of experience in the pharmaceutical industry and in biotech focused on small-molecule drugs for CNS and oncology therapeutic applications.

**Chas Bountra** is Pro-Vice Chancellor for Innovation at the University of Oxford, Professor of Translational Medicine in the Nuffield Department of Clinical Medicine, Director of the Centre for Medicines Discovery, and Professorial Fellow at Keble College, Oxford. Prior to returning to Oxford in 2008, Chas was Vice President and Head of Biology at GlaxoSmithKline. Chas is also an invited expert on several government and charitable research funding bodies, and an advisor for many academic, biotech and pharma drug discovery programs. In 2012 he was voted one of the ‘top innovators in the industry’, in 2014 he received the ‘Rita and John Cornforth Award’ from the Royal Society of Chemistry, in 2017 and 2018 he was voted ‘Master of the Bench’ from the Medicine Maker Power List, and in 2018 he was awarded the ‘Order of the British Empire’ in the New Year’s Honours List.

**Sohini Chowdhury** joined The Michael J. Fox Foundation for Parkinson's Research in 2005. Sohini was elevated to Deputy Chief Executive Officer in 2017 and expanded her responsibilities as Head of Research, steering the Foundation's grant funding and research programs in 2021. Sohini oversees the spectrum of research the Foundation is engaged in, from early-stage discovery research to clinical programs, as well as policy and advocacy. She works closely with the Foundation's executive leadership and Board of Directors in building the organization's capacity as an unprecedented stakeholder in drug development. She is a resourceful, patient-focused problem solver, whose efforts are demonstrably accelerating progress toward treatment breakthroughs and a cure.

**Julija Hmeljak** is Senior Editor at Disease Models & Mechanisms (DMM), a journal published by The Company of Biologists. DMM, as an open access biomedical journal, recognizes the privilege and the responsibility of scientific publishing in facilitating the successful translation from bench to bedside.

**Shannon Weiman** is Scientific Communications Manager at Keystone Symposia. After completing her PhD in Biomedical Sciences at the University of California, San Diego, specializing in microbiology, infectious disease and immunology, she spent over a decade as a freelance biomedical writer, working with academic, industry and government research leaders, including Stanford University, the University of California San Francisco, the FDA, the American Society for Microbiology and many others.

**Box 2. Action items**

We propose a set of action items to facilitate the discovery, development and implementation of effective brain therapeutics:

- Establish inclusive pipelines, from patients' needs to clinical proof-of-concept approaches that potentiate personalized therapeutic outcomes.
- Optimize clinical studies in which the ultimate success would be to understand the mode of action and therapeutic benefit in real-world, stratified populations. Learn from failed clinical trials and take advantage of integrated knowledge from different diseases.
- Take advantage of large-scale technological platforms and resulting data to ensure reproducibility for real innovation and efficient use of patient-derived samples from different diseases. Leverage these technologies to identify targets and pathways shared across individual diseases.
- Significant discoveries can come from anywhere. As no one institution has the expertise, funds and resources to address the challenge of clinical translation, we need to be more efficient in thinking inclusively, forging global collaborations and bringing together multiple stakeholders whose combined expertise can cover the drug discovery process to avoid inefficiency.
- Stimulate open access scientific publishing that can bring together scientists working at different stages of research and with diverse patient populations, allowing them to effectively exchange ideas, data, tools and expertise to create hubs (as briefly outlined in the point above) to foster translational neuroscience.
-   Continue funding fundamental research that deepens our understanding of the mechanism of CNS disorders, leading to diverse translational output.

### Patients: the key stakeholders

Over the past decades, our understanding of the genetic component of brain disorders has advanced dramatically. Taking PD as an example, the trajectory of knowledge and discovery in the past 20 years has been unprecedented (Bloem et al., 2021; Dhanwani et al., 2022; Anderson et al., 2022). The repertoire of treatment options evolved from attempting to merely mitigate motor symptoms, which are only one aspect of PD, to exploring the complex pathways and processes of this heterogeneous disease, with the ultimate goal of stopping the progression of motor, cognitive and sensory symptoms – therefore improving patients' quality of life. The biggest advances in this golden era of PD drug discovery have stemmed from increased patient engagement with research to help steer its direction and participation in research projects, providing a source of insights into the precise molecular pathophysiology of their disease. This patient-centered view also encourages us to re-examine large amounts of data with a big-data approach that maximizes the output of all clinical trials, both successful and failed, and respects the efforts of all patients who were willing to participate in research. Without such insights, even profound advances in technology will fail to advance the development of effective therapies.

There are, however, inherent complications when patients are involved in directing the course of research. Ensuring that a partnership between patients and researchers is meaningful and productive requires setting clear expectations of goals. Researchers must define what is and is not in the scope of a given project, and clarify the roles and responsibilities of the groups involved in the partnership. Recognizing how important it is to integrate diverse perspectives and experiences, a fruitful partnership may require clear communication of scientific background so that patients, whether very knowledgeable or new to the research and/or drug development process, can be key contributors to the project. Despite these challenges, if we keep the patients at the center of our research paradigm and balance their unique perspective with researchers' expertise, we can design better studies to maximize translational efforts.

### Bench to biotech: a critical path for collaboration

There is an urgent need to shorten the gap between basic discovery and clinical application. It is widely acknowledged that most therapeutic candidates, including those that show promise in preclinical development, ultimately fail in the clinical setting (Cowling and Thielemans, 2020). This is particularly true for complex neurological or psychiatric disorders for which animal models and associated outcomes, such as behavior, are indirect proxies for the human diseases. Inevitably, this prompts researchers to question whether they have identified the right target, dose and dosing regimen of the drug, and the correct patient (sub)group. Consequently, researchers are motivated to design their preclinical experiments and trials to address these questions. Given the limitations of classical models, the community should take advantage of preclinical biotechnological progress, such as pharmacogenomic approaches to identify new therapeutic targets and genome engineering advances for functional assays, such as conditional reporter alleles, CRISPR/Cas9, Gal4-UAS and deep mutational scanning. Additionally, embracing smaller genetically tractable model organisms, such as *Drosophila* and *Caenorhabditis elegans* (Verheyen, 2022; Kropp et al., 2021), can improve the functional annotation of genome variation in diseases, deepening our understanding of the essential cellular processes in the affected systems for efficient translation to human disease biology.

Although the tools to develop new therapies for CNS disorders have improved in recent decades, the COVID-19 pandemic has deeply affected scientific communities and research. Lockdowns have restricted access to laboratories and clinical facilities, clinicians have been focused on treating the acutely ill, and manufacturers have pivoted to supplying testing and treatments for severe acute respiratory syndrome coronavirus 2 (SARS-CoV-2) infection. Despite the disruption, the pandemic has also provided a sense of urgency and collaboration. New COVID-19 therapeutics have been developed at a dramatic speed, more than five times faster than the average time for other diseases. Although the development of vaccines and drugs has been achieved by building on previously developed assets, this confirms that collaboration is of key importance and can accelerate discovery. The COVID-19 pandemic brought a level of democratization of the scientific effort and removed many barriers in international collaboration by forcing the community to convene virtually. During this time, many datasets and analytic tools have been made freely available to the community, further enhancing accessibility and speeding up discovery.

Neuroscience stands to learn a great deal from this. Our community has demonstrated great progress as exemplified by an approved targeted treatment for AD, the anti-amyloid-β antibody aducanumab. Although further evaluation of its efficacy is required, this new treatment represents a remarkable achievement and stimulates renewed interest in brain therapies [see U.S. Food and Drug Administration (FDA) press release, 2021].

Despite this breakthrough, a major challenge for development of brain therapeutics is the heterogeneity of the diseases with respect to etiology, rate of progression and clinical outcomes. The worldwide community is working on biomarkers that could allow molecular diagnosis of the syndromic diseases. Such studies are enabled by participation of patients and their family members in natural history studies. The pace of discovery of disease state biomarkers could be hastened by collaborative research on brain diseases that may share common molecular mechanisms, like insoluble protein aggregates in PD, AD and amyotrophic lateral sclerosis (ALS) (Fare and Shorter, 2021). A number of natural history studies have been undertaken, and open source data have been made available (see, for example, Atkins et al., 2012; Cammack et al., 2019; clinical trials NCT05349019 and NCT04454892). Thus, interdisciplinary cross-disease approaches that could leverage large datasets and machine learning will help identify biomarkers and actionable therapeutic targets. In turn, this would result in precision medicine approaches to brain diseases, as has been seen in the cancer field. Optimized drug discovery pipelines leverage academic resources, attract pharmaceutical industry partners and involve practicing clinicians not only to generate and test candidate therapeutics, but to bring these to phase I and II clinical trials.

We propose that such an approach fosters dynamic innovation in drug discovery linked to patients' needs. In brain therapeutics, this implies careful stratification that can de-risk emerging therapies through early clinical proof-of-concept studies, where the right drug is tested in the patient population that is most likely to benefit from said (candidate) drug.

Open collaboration can also be potentiated in a structured manner, with public funders worldwide promoting models of public–private collaboration for novel discoveries. In the USA, one example includes the Howard Hughes Medical Institute (HHMI) Collaborative Innovation Awards, which is a pipeline-like program supporting shared project goals centered on successful individual programs that are already funded. Other examples include the Aligning Science Across Parkinson's program that encourages collaboration, resource generation and data sharing, and the National Center for Advancing Translational Sciences Clinical and Translational Science Awards Program that exemplifies team-based research to accelerate translational research. Additionally, the National Institutes of Health (NIH) Helping to End Addiction Long-term (HEAL) program in pain therapeutics spans multiple NIH Institutes and Centers and funds programs across the USA with the common goal of improving the treatment of pain while reducing the public health burden of opioid addiction.

The EU has also implemented different research infrastructures whereby publicly funded researchers can advance their research through collaboration, such as the European Research Infrastructures Consortia (ERICs) and the Innovative Medicines Initiative (IMI). Individual European countries have also developed their own programs, such as Apollo Therapeutics in the UK, ChemBioFrance or the Spanish  Kærtor Foundation public–private partnership initiatives. These joint ventures in Europe link existing institutions and companies with a common goal, remove staff mobility barriers and provide tax benefits. This reduces bureaucracy, meaning that scientists in academia and industry can focus on the science and complement each other's expertise. Furthermore, the University of Oxford joined forces with Alzheimer's Research UK, a patient-driven charity, to start a drug discovery pipeline specifically for treating dementia.

The pharmaceutical industry is also incorporating new models for innovative collaboration, such as open innovation departments or innovation centers devoted to developing the second wave of Open Innovation in drug discovery, including AstraZeneca in Cambridge and the worldwide Innovation Centers of Johnson & Johnson.

For real efficacy, public and non-profit efforts must be connected with a wide range of researchers, clinicians, regulators, industry and patients, from the beginning of the process to the end, to translate new mechanisms into first-class drugs. Such inclusive scientific consortia will remove redundancy in research, identifying truly novel targets and ultimately accelerating the discovery of effective and safe treatments.

### Communication: a key role of scientific publication

The role of scientific publishing in biomedical research has changed significantly, and we must now move on from regarding journals as mere repositories in which scientists publish their work. The advent of the COVID-19 pandemic has profoundly affected scientific communication and has pushed academic publishers to re-evaluate their roles.

In neuroscience in particular, the line of discovery from causative pathophysiology to plausible treatment is long, but open access scientific publishing can make this process less tortuous. Journals can achieve this by acting as a hub to bring scientists together, allowing them to effectively exchange ideas, data, tools and expertise. By encouraging the publication of commentary- and correspondence-type articles that discuss the latest research, and by including peer review histories with published research articles, journals can become a forum in which the broader community shares expertise and opinion linked to specific discoveries. This forum function can also transcend the written format by linking journals with in-person and virtual gatherings, such as panel discussions, conferences, webinars and workshops.

An additional way in which publishers can ease the progression from basic discovery to the clinic is by publishing validation studies and negative results, and by offering authors solid ‘scoop’ protection policies. Furthermore, removing paywalls renders this knowledge – created both by work at the bench and by placing this work in the broader context via commentaries and discussion – accessible to everyone. This empowers patients to improve their own understanding of their disease and take an active part in scientific discovery.

The expansion of preprint servers and the broader adoption of preprints gives researchers fast access to the most novel findings, paving the way for new hypotheses and more open scrutiny of research findings. Despite these advantages, it is important to remember that preprints are not vetted in a conventional editorial and peer review process, which means that they should be regarded as a crucial step in the knowledge dissemination journey, not its endpoint. In short, we stress that preprints should foster essential critical discussion of the data and appeal to readers to avoid ‘peer-review by tweet’.

These important changes in scientific publishing require an open discussion about best practices, ethics and responsible conduct, and new approaches to assess research. Although some of these conversations may not be easy, they will ensure that journals are truly supporting researchers in academia and in industry, and are potentiating scientific progress in an inclusive manner.

### Funders: the key questions

Accelerating drug discovery in neuroscience via new strategies for collaboration also requires a new vision of funding. Risk of failure, time and costs are serious limiting factors for investment in brain therapeutics. Despite its questionable efficacy, the recent approval of aducanumab as the first targeted treatment for AD has renewed an appetite for neuroscience and neurodegeneration research. However, many aspects of the translational research funding system still need to be challenged.

Funding agencies were historically more supportive of safer, consensus-driven research rather than of disruptive and risky developments. Tackling COVID-19 has shown that it is possible to shorten the time from initial discovery to pioneering clinical trials, assuming a higher attrition rate at basic and preclinical stages. This optimizes expectations and reduces patient burden and costs once therapies reach clinical trials.

The inclusive translational pipelines we discuss in this article leverage academic resources, involve practicing clinicians, and attract pharmaceutical industry and venture partners. However, it is important to note that any such collaborative platforms should include the high-quality basic science of individual investigators, which is the foundation for the progress of therapeutics as it enables the evolution and testing of hypotheses that arise from scientific discovery. Therefore, it is important to diversify the funds enough so that fundamental research is maintained. Importantly, studies within such collaborative arrangements need to be executed with suitable adherence to the highest scientific and ethical standards if they are to provide meaningful patient benefits.

Providing patient material and clinical information is key to identifying therapeutic windows across a disease's spectrum, from the very earliest to the very advanced stages that have devastating effects on a patient's quality of life, but this process requires significant resources. The Parkinson’s Progression Markers Initiative (PPMI), a natural history study sponsored primarily by The Michael J. Fox Foundation for Parkinson’s Research, with participation from other industry and non-profit partners, illustrates this point. It began in 2010, collecting significant clinical imaging data along with corresponding biosamples, for data analysis and biomarker development.

Taken together, the progression of a novel therapeutic towards clinical use requires commitment from all partners, with a new vision in funding models to ensure that this energy results in concrete progress for patients.

## Conclusions

**Fig. 1.**
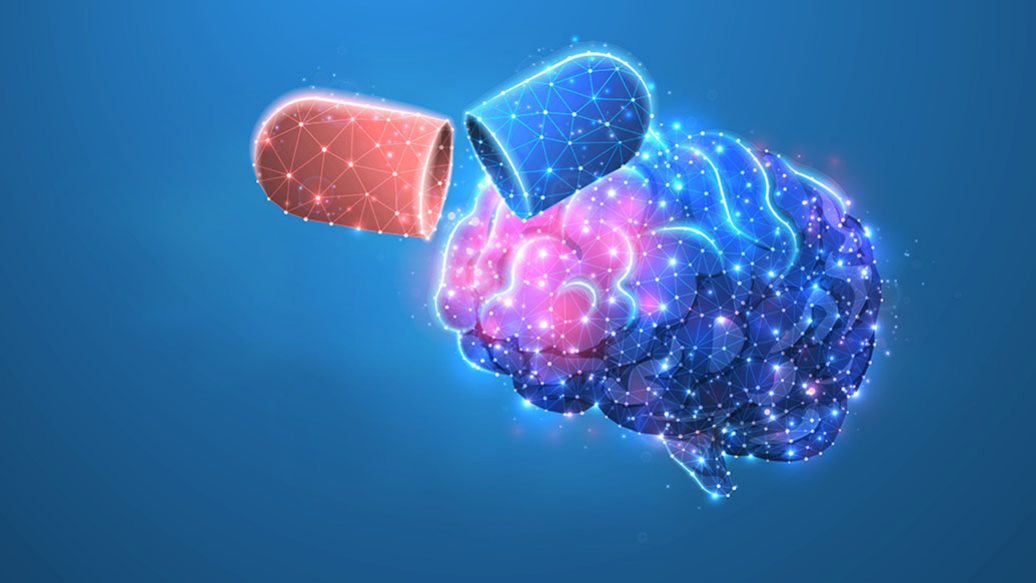
**Progress in brain therapeutics requires collaboration and a rethink of existing drug discovery networks.** This illustration was originally used in the promotional materials for the ‘Brain Therapeutics: Lessons Learned from Pandemic Times’ virtual panel organized by Keystone Symposia. Image courtesy of Keystone Symposia. This image is not published under the terms of the CC-BY license of this article. For permission to reproduce, please contact Keystone Symposia.

The way that the COVID-19 pandemic has rallied scientists is a unique example rather than a precedent-setting event. Nevertheless, it has taught us how much the community can achieve when barriers are removed and community motivation is exceptionally high. Vaccine development, as a whole, has greatly benefitted from this pandemic, as have broad mRNA research and drug delivery technologies (Bhat et al., 2021). By contrast, other fields, and clinical trials in particular, have slowed down due to limited patient enrolment. Although neuroscience has borne some of this cost, it is now returning to the forefront because the neurological sequelae of acute COVID-19 are emerging. Additionally, clinical successes like the extended survival of patients with diseases that do not primarily arise in the CNS, such as cancer and muscular disease, allow researchers to explore the chronic effects of these diseases on the CNS, bringing brain research and therapeutics closer to other fields. We feel a renewed sense of urgency in applying the lessons learned in advancing brain disease research to a more effective and safe therapeutic for patients (Fig. 1). We invite the readers to share our enthusiasm for this progression.
